# Clinical relevance of circulating tumor DNA assessed through deep sequencing in patients with metastatic colorectal cancer

**DOI:** 10.1002/cam4.1913

**Published:** 2018-12-21

**Authors:** Hiroki Osumi, Eiji Shinozaki, Yoshinori Takeda, Takeru Wakatsuki, Takashi Ichimura, Akio Saiura, Kensei Yamaguchi, Shunji Takahashi, Tetsuo Noda, Hitoshi Zembutsu

**Affiliations:** ^1^ Department of Gastroenterology, Cancer Institute Hospital Japanese Foundation for Cancer Research Tokyo Japan; ^2^ Department of Hepato‐Biliary‐Pancreatic Surgery, Cancer Institute Hospital Japanese Foundation for Cancer Research Tokyo Japan; ^3^ Department of Medical Oncology, Cancer Institute Hospital Japanese Foundation for Cancer Research Tokyo Japan; ^4^ Cancer Precision Medicine Center, Cancer Institute Japanese Foundation for Cancer Research Tokyo Japan

**Keywords:** circulating tumor DNA (ctDNA), liquid biopsy, metastatic colorectal cancer, *RAS*, tumor burden

## Abstract

Because circulating tumor DNA (ctDNA) studies focusing on only one or a few genes to monitor the disease progress or treatment response are unlikely to find its clinical significance, the development of cell‐free DNA (cfDNA) panel covering hundreds of mutation hot spots is important for the establishment of clinically practical ctDNA detection system. We enrolled 101 patients with metastatic colorectal cancer (mCRC) who received chemotherapy. Amplicon‐based genomic profiling of 14 genes, which are commonly mutated in CRC, in plasma by next‐generation sequencing (NGS) was carried out to evaluate the feasibility of this assay and was compared with their clinical parameters and *RAS* status in matched tissue samples. Somatic mutations of the 14 genes in plasma cfDNA were detected in 88 patients (87.1%) with mCRC. Mutations in *TP53*, *KRAS*, and *APC* genes were detected in 70 (69.3%), 39 (38.6%), and 24 (23.7%) patients, respectively. Mutant allele frequencies in plasma were significantly associated with metastasis (liver, *P* = 0.00004, lymph node, *P* = 0.008, number of metastatic organs, *P* = 0.0006), tumor markers (CEA, *P* = 0.000007, CA19‐9, *P* = 0.006, LDH, *P* = 0.00001), and tumor diameter (maximum, *P* = 0.00002, sum of diameter, *P* = 0.00009). The overall concordance rate of *RAS* status between ctDNA and matched tissue was 77.2% (78/101). Our data confirmed that mutant allele in cfDNA can be sensitively detected by amplicon‐based NGS system. These results suggest that ctDNA could be a novel diagnostic biomarker to monitor changes in mutational status and tumor burden in patients with mCRC.

## 1 INTRODUCTION

Liquid biopsy, which is based on the analysis of circulating tumor DNA (ctDNA), circulating tumor cells (CTCs), and exosomes secreted from cancer cells in peripheral blood, has been expected to enable us to characterize the cancer genome by minimally invasive methods for patients with cancer.^1^, ^2^ Of them, ctDNA is one of the most well‐studied technologies because of the recent development of high‐sensitivity next‐generation sequencer and the ability to comprehensively characterize the cancer cells and to detect the time‐course change in tumor genotype.^3^, ^4^, ^5^

The clinical application of ctDNA detection as a “liquid biopsy” has been studied and reported.^6^, ^7^ ctDNA is fragmented DNA released from cancer cells into the blood. It represents a small fraction of cell‐free DNA (cfDNA), which is thought to be released into the blood as a result of cell apoptosis and/or necrosis.^7^, ^8^, ^9^ ctDNA is thought to carry information from the entire tumor genome and provide insight into clonal heterogeneity and evolution of cancers.^10^, ^11^ Although ctDNA are cleared from the blood by the liver and kidney, its half‐lives range from 15 minutes to several hours, suggesting that it could be a real‐time biomarker for assessment of quality (tumor genotype) and quantity (tumor burden) of the cancer.^7^, ^12^ Recently, Food and Drug Administration approved epidermal growth factor receptor (*EGFR*) mutation test using plasma as a diagnostic tool for the detection of *EGFR* mutations to predict the erlotinib (EGFR tyrosine kinase inhibitor) response in patients with non‐small‐cell lung cancer.^13^, ^14^ Thus, ctDNA monitoring could be useful biomarker for tumor recurrence, drug resistance, and treatment response, which enable physicians to select more appropriate treatment to each patient.^15^, ^16^, ^17^

Techniques for detection of small amount of mutant allele in plasma such as digital PCR (dPCR) or combination of emulsion dPCR and flow cytometry (BEAMING) have had superior sensitivity to the other methods.^18^, ^19^, ^20^ However, studies analyzing ctDNA to monitor the disease progress or treatment response are likely to focus on only one or a few genes using dPCR and showed its limited clinical significance.^21^, ^22^, ^23^ Hence, the development of cfDNA panel, which covers mutation hot spots of commonly mutated genes in CRC, is needed to establish high‐sensitive diagnostic system for plasma ctDNA detection in patients with CRC. In this study, we explored the feasibility of targeted NGS cfDNA panel for 14 genes frequently mutated in CRC using 101 plasma cfDNA of patients with mCRC and investigated its clinical utility by analyzing the relationship between plasma ctDNA and clinicopathological factors.

## 2 MATERIALS AND METHODS

### 2.1 Patients

The primary endpoint of this study was to evaluate the feasibility of the ctDNA analysis for detection of prevalent mutations in CRC. One hundred and one patients with mCRC, who received chemotherapies at Cancer Institute Hospital, Japanese Foundation for Cancer Research, were consecutively enrolled in this study from February to June 2017. As shown in Table 1, a specific course of treatment was not required for enrollment in this study. Union for International Cancer Control (UICC) TNM classification was used to determine the tumor and nodal status. This study was approved by the Institutional Review Boards of the Japanese Foundation for Cancer Research (Tokyo, Japan). Written informed consent was obtained from all patients.

**Table 1:** Patient demographics and clinical characteristics

Characteristics	Total (N = 101) no. of patients (%)
Age at enrollment, y	
Median [range]	64 [30‐84]
Gender	
Male	63 (62.3)
Female	38 (37.7)
Treatment line at the time of sampling	
Neoadjuvant chemotherapy	12 (11.9)
1st line	37 (36.6)
2nd line	27 (26.7)
3rd or later line	17 (16.9)
Adjuvant chemotherapy	8 (7.9)
Treatment at registration at the time of sampling	
FOLFIRI/CPT‐11 + anti‐VEGF antibody	38 (37.6)
SOX/CapeOX/FOLFOX/FOLFOXIRI + anti‐VEGF antibody	18 (17.8)
FOLFOX + anti‐EGFR antibody	15 (14.8)
FOLFIRI/CPT‐11 + anti‐EGFR antibody	10 (9.9)
FOLFOX	5 (4.9)
Regorafenib	4 (4.0)
CapeOX	3 (3.0)
TAS102	3 (3.0)
5‐FU + LV/Capecitabine + anti‐VEGF antibody	2 (2.0)
TAS102+anti‐EGFR antibody	2 (2.0)
Capecitabine	1 (1.0)
Primary site	
Right‐sided colon	24 (23.8)
Left‐sided colon	77 (76.2)
Resection of primary tumor	
Yes	68 (67.3)
No	33 (32.7)
Metastatic site	
Single organ	43 (42.6)
Multi‐organ	58 (57.4)
Liver	75 (73.5)
Lung	41 (40.5)
Lymph node	32 (31.6)
Peritoneum	21 (20.7)
Others	12 (11.8)
*RAS* status in tissue	
Wild‐type	60 (59.4)
Mutant	41 (40.6)
Prior Chemotherapy regimen	
Anti‐VEGF antibody	76 (75.2)
Anti‐EGFR antibody	41 (40.6)
Cytotoxic drug(s) only	4 (3.96)
Tumor markers	
CEA median, [range]	16 [1‐7479]
CA19‐9 median, [range]	25 [2‐≥50 000]

CapeOX, a combination of capecitabine with oxaliplatin; CA19‐9, carbohydrate antigen 19‐9; CEA, carcinoembryonic antigen; CPT‐11, irinotecan hydrochloride hydrate; EGFR, epidermal growth factor receptor; FOLFIRI, a combination of calcium folinate and fluorouracil with irinotecan hydrochloride hydrate; FOLFOX, a combination of calcium folinate and fluorouracil with oxaliplatin; FOLFOXIRI, a combination of calcium folinate and fluorouracil and irinotecan hydrochloride hydrate with oxaliplatin; 5‐FU, fluorouracil; LV, calcium folinate; *RAS*, rat sarcoma viral oncogene homolog; SOX, a combination of tegafur, gimeracil, oteracil potassium with oxaliplatin; TAS102, trifluridine, tipiracil hydrochloride; VEGF, vascular endothelial growth factor.

### 2.2 Blood samples, ctDNA isolation and sequencing

Blood samples were collected into EDTA tubes following the manufacturer’s instructions. Plasma from blood sample was obtained by centrifugation at 1600 *g* for 10 minutes at 4°C, followed by another spin at 16 000 *g* for 10 minutes at 4°C to remove cellular debris. cfDNA was extracted from 2 mL plasma using a MagMAX cfDNA Isolation Kit (Thermo Fisher Scientific) following the manufacturer’s instructions. Oncomine Colon cfDNA Assay (Thermo Fisher Scientific) was used to generate libraries from cfDNA following the manufacturer’s instructions. Quality control of the libraries was performed using the Qubit^®^2.0 and 2100 Bioanalyzer (Agilent Technologies). Ion Chef™ System and Ion 530™ Kit‐Chef were used for template preparation, followed by sequencing on Ion S5 system using Ion 530 chips. Six‐plex library pool was applied on an Ion 530 chip. The cfDNA panel, which covers 14 genes with >240 hot spots (SNVs and short Indels), such as *AKT1*,* BRAF*,* CTNNB1*,* EGFR*,* ERBB2*,* FBXW7*,* GNAS*,* KRAS*,* MAP2K1*,* NRAS*,* PIK3CA*,* SMAD4*,* TP53*, and *APC*, was used in this study. The clean reads were mapped to the human reference genome (hg19). Variant caller was used to filter and call the mutations in targeted regions in each gene.^24^, ^25^ Cutoff level for each mutant allele frequency was defined by “variant caller” for each sample (patient). The range of the limit of detection for the variants in *KRAS* and *NRAS* was from 0.05 to 0.20 in this study.

### 2.3 Tumor tissue DNA sequencing

Genomic DNA was extracted from fixed paraffin‐embedded tissues obtained from biopsies or surgical resections. DNA extraction was performed using a modified protocol as described previously.^26^, ^27^ For tissue *KRAS* and *NRAS* test, RASKET KIT (MBL), which applies the Polymerase Chain Reaction‐Reverse Sequence‐Specific Oligonucleotide method (PCR‐rSSO), was used following the manufacturer’s protocol. We examined twelve types of *RAS* exon 2 (G12S, G12C, G12R, G12D, G12V, G12A, G13S, G13C, G13R, G13D, G13V, and G13A), eight types of *RAS* exon 3 (A59T, A59G, Q61K, Q61E, Q61L, Q61P, Q61R, and Q61H), and four types of *RAS* exon 4 (K117N, A146T, A146P, and A146V) mutations using Luminex 100/200TM (Luminex) and UniMAG (MBL) system, as previously described.^28^, ^29^

### 2.4 Statistical analysis

We investigated the association of clinicopathological factors with ctDNA levels, which are the highest allele frequency of the detected mutant alleles in each patient, and amount of cfDNA using Welch’s *t* test. The differences in sum of the tumor diameter among *RAS* status groups were also evaluated by Welch’s *t* test. Statistical tests provided two‐sided *P* values, and a significance level of *P* < 0.05 was used. Statistical analyses were carried out using statistical software “EZR” (Easy R), which is based on R and R commander.

## 3 RESULTS

### 3.1 Patient characteristics

To examine the feasibility of ctDNA detection in plasma using amplicon‐based NGS, we recruited 101 patients with mCRC receiving chemotherapy in neoadjuvant, adjuvant, or metastatic setting. The characteristics of these 101 patients who were diagnosed to have mCRC were summarized in Table 1. Their median age at the time of recruitment was 64 years old (range, 30‐84 years), and 63 were men (62.3%). Liver was the most frequent site of metastasis (73.5%), followed by lung (40.5%), lymph node (31.6%), and peritoneum (20.7%). Of the 101 patients, 60 (59.4%) showed wild‐type *RAS* in their tissues obtained from tissue biopsy or surgical specimen, and 41 (40.6%) patients received anti‐EGFR antibody therapy before blood collection for ctDNA analysis (liquid biopsy) in this study (Table 1).

### 3.2 Detection of somatic mutations in plasma

**Figure 1:**
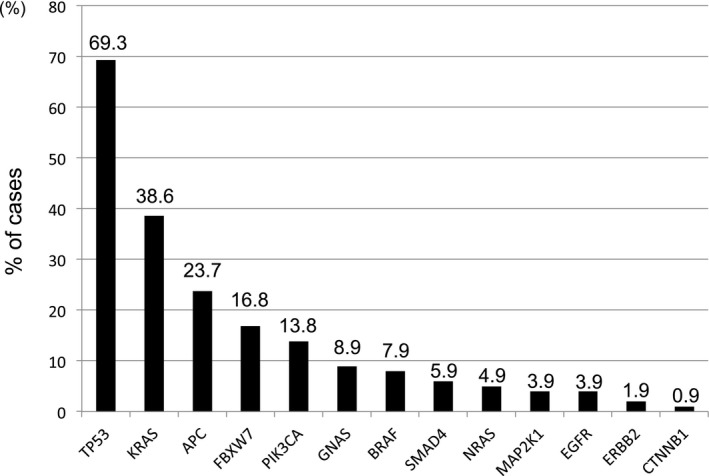
Frequencies of mutated genes in 101 CRC patients’ plasma. One or more mutations were detected in all genes on the panel except *AKT1*

Of the 101 patients recruited in this study, one or more somatic mutations in the 14 colorectal cancer‐related genes (*AKT1*,* BRAF*,* CTNNB1*,* EGFR*,* ERBB2*,* FBXW7*,* GNAS*,* KRAS*,* MAP2K1*,* NRAS*,* PIK3CA*,* SMAD4*,* TP53*, and *APC*) were detected in 88 (87.1%) plasma of patients with mCRC (Figure S1). The range of the mutant allele frequencies in each gene was shown in Table S1. Mutations in *TP53*, *KRAS*, and *APC* genes were detected in 70 (69.3%), 39 (38.6%), and 24 (23.7%) patients, respectively (Figure 1). *FBXW7* and *PIK3CA* genes were also frequently mutated (17 (16.8%) and 14 (13.8%) patients, respectively, Figure 1). Mutations in *GNAS* (8.9%), *BRAF* (7.9%), *SMAD4 *(5.9%), *NRAS* (4.9%), *MAP2K1* (3.9%), *EGFR* (3.9%), *ERBB2* (1.9%), and *CTNNB1* (0.9%) were less common (<10% of patients) compared to the above genes (Figure 1).

### 3.3 Association of clinical factors with cfDNA in patients with mCRC

To assess the clinical utility of mutation detection in plasma of patients with mCRC by using amplicon‐based deep sequencing, we investigated the association between clinical factors, which could be correlated with tumor burden, and ctDNA level (the highest allele frequency of the detected mutant alleles in each patient) in plasma of patients with mCRC. The clinical factors used in this association study were primary tumor location, metastatic target organ, number of metastatic organs, tumor markers, and tumor diameters (Table 2). Patients with liver or lymph node metastasis showed significantly higher ctDNA level in their plasma compared to those without them (*P* = 0.00004 and *P* = 0.008, respectively, Table 2). Furthermore, ctDNA levels of patients with multi‐organ metastasis were significantly higher than those with single organ metastasis (*P* = 0.0006, Table 2). Tumor markers, carcinoembryonic antigen (CEA), carbohydrate antigen 19‐9 (CA19‐9), lactate dehydrogenase (LDH), were significantly associated with ctDNA level of patients with mCRC (*P* = 0.000007, *P = *0.006 and *P* = 0.00001, respectively, Table 2), and maximum and sum of the tumor diameter were also significantly associated with their ctDNA level (*P* = 0.00002 and *P* = 0.00009, Table 2). On the other hand, total amount of cfDNA was significantly associated with only CEA, maximum and sum of the tumor diameter, and the association was weaker (*P* = 0.002, 0.009 and 0.004, respectively) than those observed in ctDNA level.

**Table 2:** Association of clinical factors with ctDNA level and amount of cfDNA in plasma

Clinical characteristics	ctDNA level^a^ (average, %)	*P *value	cfDNA (ng/ml)	*P *value
Primary tumor location				
Right‐sided colon	15.4	0.18	4.2	0.10
Left‐sided colon	8.6		2.1	
Liver metastasis				
Positive	13.1	0.00004	2.9	0.06
Negative	2.0		1.7	
Lung metastasis				
Positive	10.5	0.89	2.0	0.17
Negative	10.1		3.0	
Peritoneal metastasis				
Positive	10.5	0.94	3.9	0.10
Negative	10.2		2.2	
Lymph node metastasis				
Positive	19.0	0.008	2.7	0.87
Negative	6.2		2.5	
Number of metastatic organs				
Single organ	3.7	0.0006	2.5	0.80
Multi‐organ	14.8		2.7	
CEA (ng/mL)				
<5	1.4	0.000007	1.3	0.002
≥5	13.5		3.0	
CA19‐9 (U/mL)				
<37	5.8	0.006	2.0	0.08
≥37	16.2		3.4	
LDH (U/L)				
<245	2.5	0.00001	2.1	0.17
≥245	20.4		3.2	
D‐dimer (μg/L)				
<1.6	10.2	0.63	2.3	0.15
≥1.6	12.7		4.6	
Maximum tumor diameter^b^ (mm)				
<median^c^	3.3	0.00002	1.6	0.009
≥median	18.4		3.5	
Sum of the tumor diameter^b^ (mm)				
<median^d^	2.5	0.00009	1.5	0.004
≥median	17.2		3.8	

CA19‐9, carbohydrate antigen 19‐9; CEA, carcinoembryonic antigen; LDH, lactate dehydrogenase.

a The highest allele frequency of the detected mutant alleles in each patient.

b RECIST ver 1.1 criteria.

c 28 mm.

d 46 mm.

### 3.4 Comparison of RAS status between paired tissue and plasma samples

In this study, tissue biopsy had been carried out prior to blood collection for plasma ctDNA analysis (liquid biopsy), and all patients have received anticancer therapy including chemotherapy and/or molecular targeted therapy between tissue and liquid biopsy. To investigate the change in *RAS* status after the chemotherapy and/or molecular targeting therapy, we compared the *RAS* status in tumor obtained by tissue biopsy with those in plasma (ctDNA). Of the 101 patients used in this study, 41 patients (40.6%) had a tissue *RAS* mutation, which were analyzed by PCR‐reverse sequence‐specific oligonucleotide (PCR‐rSSO, RASKET^®^) as shown in Table 3. Of them, plasma *RAS* mutation was also found in 31 patients (75.6%). On the other hand, 60 patients (59.4%) did not have a tissue *RAS* mutation, and of them, 47 patients (78.3%) also showed no mutated *RAS* in their plasma (Table 3). Hence, the overall concordance rate of *RAS* status between tissue and ctDNA in 101 patients was 77.2% (78/101), indicating that 23 patients showed discordant mutation status of *RAS* between tissue and matched plasma (Table 3). We further investigated the cause of the discordant *RAS* status between tissue and plasma. Of the 13 patients, who had no *RAS* mutation in tissue but have the mutation in plasma, 11 patients (84.6%) had received anti‐EGFR inhibitor therapy before liquid biopsy (Table 4). However, of the 47 patients, who had no *RAS* mutation in both tissue and plasma, only 29 patients (61.7%) had received anti‐EGFR inhibitor therapy before liquid biopsy (Table S2), suggesting the impact of anti‐EGFR inhibitor therapy on change in *RAS* status. On the other hand, sum of the tumor diameter of patients with *RAS* mutation‐positive in tissue but negative in plasma (Table 4) was significantly smaller than those of patients with *RAS* mutation‐positive in both tissue and plasma (Table S2), suggesting that decrease in tumor burden causes undetectable *RAS* mutation in plasma (16.2 and 86.0 mm on average, respectively, *P* = 0.00000015, Figure S2).

**Table 3:** *RAS* mutations detected in paired tissue and plasma

	Tissue *RAS*		
	No mutated	Mutated	Total
Plasma *RAS*			
No mutated	47	10	57
Mutated	13	31	44
Total	60	41	101

*RAS*, rat sarcoma viral oncogene homolog.

**Table 4:** Characterization of cases showing discordant *RAS* status between tissue and plasma

No mutated *RAS* in tissue, mutated *RAS* in plasma							
No	Codon (Plasma)	Mutant allele frequency in plasma (%)	Primary tumor resection	Site of metastasis	Chemotherapy at the time of liquid biopsy	Sum of the tumor diameter^a^ (mm)	Anti‐EGFR inhibitor therapy (before or at the time of liquid biopsy)
1	*KRAS* C/T position 25398282	0.07	+	Liver, Lung, Peritoneal	CPT‐11 + C‐mab	234	+
2	*KRAS* T/A position 25380275	2.03	+	Liver, Peritoneal, Lymph node	CPT‐11 + C‐mab	53	+
3	*KRAS* G12D	1.42	−	Liver, Peritoneal	TAS102+P‐mab	68	+
4	*KRAS* G12A, G12D	6.53, 2.97	+	Liver, Lymph node, renal	Regorafenib	144	+
5	*KRAS* G12V	0.34	+	Lung, Liver	5‐FU/LV+BEV	17	−
6	*KRAS* G12D	0.15	+	lung	FOLFOX+P‐mab	6	+
7	*KRAS* G12D, G13D	0.09, 0.14	+	Lung, Lymph node	FOLFOX+C‐mab	11	+
8	*KRAS* G12D, G13D	0.09, 0.09	+	None	CapeOX	0	−
9	*KRAS* G12D	0.13	+	Lung	FOLFOX+P‐mab	7	+
10	*KRAS* G12A, *KRAS* G12D, *KRAS* Q61H	7.45, 0.21,0.93	+	Liver	Regorafenib	92	+
11	*KRAS* G12V	0.1	−	Liver, Lymph node	FOLFOX+C‐mab	46	+
12	*NRAS* Q61K	0.22	−	Liver, Lymph node	FOLFIRI+RAM	50	+
13	*KRAS* G13D	0.37	+	Liver lung	FOLFIRI+RAM	68	+

Mutated *RAS* in tissue, no mutated *RAS* in plasma							
No	Codon (Tissue)	Mutant allele frequency in plasma (%)	Primary tumor resection	Site of metastasis	Chemotherapy at the time of liquid biopsy	Sum of the tumor diameter^a^ (mm)	Anti‐EGFR inhibitor therapy (before or at the time of liquid biopsy)
1	*KRAS* G12R	–	+	Liver	FOLFIRI+BEV	23	−
2	*NRAS* Q61K	–	+	Lung	FOLFIRI+BEV	14	−
3	*KRAS* G12V	–	+	Lung	FOLFIRI+BEV	35	−
4	*KRAS* G12D	–	+	Liver, Lymph node	FOLFOX+BEV	27	−
5	*KRAS* G12V	–	+	Peritoneal	FOLFIRI+BEV	0	−
6	*NRAS* Q61H	–	−	Liver	FOLFOX+BEV	32	−
7	*NRAS* Q61H	–	+	Lung, Lymph node	FOLFIRI+BEV	10	−
8	*KRAS* G12D	–	+	Liver, Peritonial	FOLFIRI+BEV	7	−
9	*KRAS* G13D	–	+	None	FOLFOX	0	−
10	*KRAS* G12V	–	+	Lung	FOLFIRI+BEV	14	−

BEV: bevacizumab; CPT‐11: irinotecan hydrochloride hydrate; C‐mab: cetuximab; EGFR: epidermal growth factor receptor; FOLFOX: a combination of calcium folinate and fluorouracil with oxaliplatin; FOLFIRI: a combination of calcium folinate and fluorouracil with irinotecan hydrochloride hydrate; FOLFOXIRI: a combination of calcium folinate and fluorouracil and irinotecan hydrochloride hydrate with oxaliplatin; 5‐FU: fluorouracil; LV: calcium folinate; P‐mab: panitumumab; *RAS*: rat sarcoma viral oncogene homolog; RAM: ramucirumab; CapeOX: a combination of capecitabine with oxaliplatin; SOX: a combination of tegafur, gimeracil, oteracil potassium with oxaliplatin; TAS102: trifluridine, tipiracil hydrochloride.

a RECIST ver 1.1 criteria.

## 4 DISCUSSION

In this study, we used a 14‐gene panel, which was designed by identifying frequently mutated genes in colon cancer, for deep sequencing by targeted NGS system, and presented evidence for the feasibility and clinical utility of ctDNA detection for the management of patients with mCRC. Similar to the results of the previously reported studies,^30^, ^31^ we also observed highly sensitive detection of ctDNA in plasma of patients with mCRC by using the amplicon‐based deep sequencing with molecular barcodes (Figure S1), suggesting that the panel of 14 genes with >240 hot spots selected as frequently mutated genes in colon cancer could be feasible for liquid biopsy in patients with CRC.

Although the amount of cfDNA has been reported to be associated with the clinical factors, which were suggestively correlated with tumor burden such as metastasis and tumor markers,^32^, ^33^ we found that ctDNA level is likely to be more correlated with those factors than cfDNA (Table 2). We observed significant association of ctDNA level with liver metastasis and sum of the tumor diameter in metastatic sites, which were commonly reported to be strongly associated with ctDNA level.^20^, ^34^, ^35^ While the controversial or null association of ctDNA level with the lung, lymph node and peritoneal metastasis, tumor markers, primary tumor location, and number of metastatic organs has been reported,^20^, ^34^, ^36^, ^37^ we observed significant association between ctDNA level and lymph node metastasis, number of metastatic organs, and tumor markers (CEA, CA19‐9, and LDH). Although different sample size and sampling bias may cause these discordant results, the above associations should be further validated by using a larger number of samples.

Because EGFR signaling has been recognized as an important player in CRC initiation and progression,^38^, ^39^, ^40^ EGFR inhibitors, which are effective in CRC harboring wild‐type *RAS*, have been used as one of the important molecular targeted therapies for CRC.^41^, ^42^, ^43^ Hence, *RAS* mutation analysis using plasma sample is expected to be applicable for the prediction of response to EGFR inhibitors and monitoring the change in *RAS* status of mCRC. Although the timing of liquid biopsy (blood collection) after tissue biopsy in each patient was inconsistent in this study, the concordance rate of *RAS* status between tissue and matched plasma was 77.2% (78/101). Of the remaining 23 patients showing discordant *RAS* status, plasma *RAS* mutation was not detected in 10 patients with *RAS* mutant tumors. This discordant result could be partly explained by the fact that sum of the tumor diameter in patients with tissue *RAS* mutant but without plasma *RAS* mutant (Table 4 (lower)) is significantly smaller than those in patients with mutant *RAS* in both tissue and plasma (Table S2), suggesting that mutant *RAS* was undetectable in plasma due to tumor shrinkage by the efficacy of anticancer therapy following tissue biopsy (Figure S2). In contrast, ctDNA of 13 patients showed the presence of *RAS* mutations that were not detected on tissue, and they were likely to have received anti‐EGFR inhibitor therapy before or at the time of liquid biopsy (11 cases; 84.6%, Table 4 (upper)) compared to those without *RAS* mutation in both plasma and tissue (29 out of 47 cases; 61.7%, Table S2).^44^ As one of the mechanisms of acquiring resistance to anti‐EGFR therapy for CRC, point mutation in extracellular domain of EGFR (S492R) has been reported to make resistant to this therapy,^45^, ^46^ and amplification of receptor tyrosine kinase genes such as *ERBB2* or *MET* is also associated with acquiring resistance to anti‐EGFR inhibitor.^47^, ^48^ Furthermore, resistance to anti‐EGFR inhibitors is related to constitutive activation of signaling pathways downstream of EGFR including *KRAS*, *NRAS*, and *BRAF*.^49^, ^50^ Of them, one of the most common molecular mechanisms that drive acquiring resistance to anti‐EGFR inhibitor therapy for CRC is mutations in *KRAS*.^18^, ^21^ Although we could not investigate the relationship between the emergence of *KRAS* mutation in plasma and clinical response in this study, the plasma *RAS* mutations newly emerged after this therapy in the above 11 cases may represent a sign of expansion of resistant clones to anti‐EGFR inhibitors. One of the other possibilities which causes the above discordant *RAS* status between tissue and plasma is that the deep sequencing of cfDNA used for *RAS* mutational analysis could have higher sensitivity compared to the PCR‐rSSO techniques used in *RAS* mutation analysis for tissues. Moreover, these discordant results might suggest that liquid biopsy (ctDNA analysis) could provide more comprehensive information of mutational character of tumors, therefore could avoid the influence of tissue sampling bias in *RAS* mutation test.

Because total number of ctDNA genomic alterations might be related to the characteristics of the cancers,^30^, ^51^ we examined the association of the number of mutations with metastasis, tumor markers, and sum of the tumor diameter. We observed that CA19‐9 and sum of the tumor diameters were significantly higher in patients with 2 or more mutations detected in their plasma compared with those with one mutation (CA19‐9: *P* = 0.010, diameter: *P* = 0.016, Table S3), and liver metastasis also indicated marginal association with the number of mutations (*P = *0.066, Table S3). We further examined the relationship between specific gene mutation and metastatic site and found that mutations in *KRAS*, *GNAS*, and *SMAD4* were significantly associated with lung metastasis (*P* = 0.039, 0.029, and 0.039, respectively, Table S4). Because *KRAS* mutational status could partially determine the biological aspect in colon cancer,^52^ we compared the mutations detected in plasma and clinicopathological parameters in patients with *KRAS* mutation‐positive with those in patients with *KRAS *mutation‐negative. The frequencies of lung metastasis, mutations in *TP53*, *NRAS*, and *EGFR*, the level of tumor markers (CEA and CA19‐9), and sum of the tumor diameter in patients with plasma *KRAS* mutation‐positive were higher compared to those in patients with *KRAS* mutation‐negative (Table S5).

There are limitations in this study because the number of patients assessed is limited and the detection method for *RAS* mutation is different between tissue and plasma. The use of a blood samples collected at different time points also precluded appropriate comparison of *RAS* mutational status between tissue and plasma, and single‐point blood sampling makes it impossible to analyze time‐course change in ctDNA during chemotherapies, which could be more useful information to assess the chemotherapeutic response. Moreover, the frequencies of mutated genes in plasma ctDNA of patients with CRC in our study were inconsistent with those in tissue DNA which had been reported in mutation database including The Cancer Genome Atlas.^53^, ^54^ Although the mutation frequency of *APC* gene in CRC tissue has been reported to be ~80%,^53^, ^55^ it revealed only 23.7% in our data (plasma), which was significantly lower frequency than that in tissue. The difference of the above frequencies could be partially explained by insufficient coverage of mutation detection in *APC* gene of the panel used in our ctDNA study. The further technical improvement of the panel, which covers sufficient hot spot mutations of APC gene, would increase the mutation detection of APC gene in plasma of patients with CRC. Moreover, although the limit of detection of the variant used in this study was reported to be 0.1%,^56^ we did not evaluate the detection limit of the assay in our samples. Detection limit of each mutant allele frequency was defined by the algorism in “variant caller,” which is the software for detection of variants, for each patient. Further study to evaluate the actual detection limit under several conditions (methods of blood collection, amounts of cfDNA for library construction, etc) might be needed for clinical application of this assay.

In conclusion, our data confirmed that small fraction of mutant allele in cfDNA could be detected by amplicon‐based NGS assay using cfDNA panel. Although our data suggested that the quantification of ctDNA by using NGS with molecular barcode system could be a novel biomarker for tumor burden, further technical development to increase sensitivity and specificity for detection of mutant allele and robust clinical test would be needed for the development of clinical practice recommendation.

## CONFLICT OF INTEREST

None of the authors have any conflicts of interest to declare.
