# Supplementary figures and images for: Clinical relevance of circulating tumor DNA assessed through deep sequencing in patients with metastatic colorectal cancer
Source: Cancer Med. 2018 Dec 21;8(1):408–17. doi: 10.1002/cam4.1913 (PMC6346227; doi:10.1002/cam4.1913)

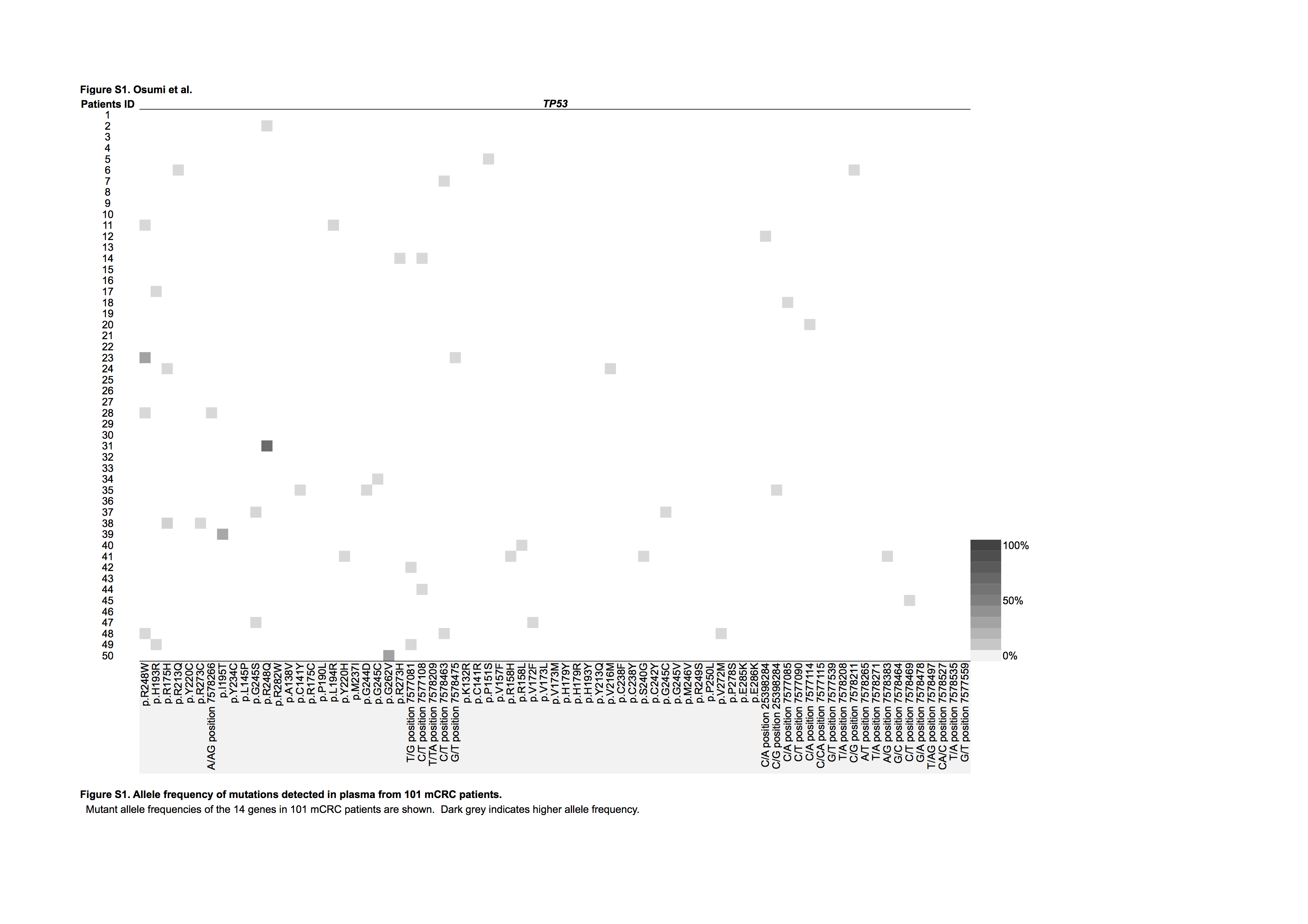

Supplement: Supplementary file 1 [file CAM4-8-408-s001.tiff]

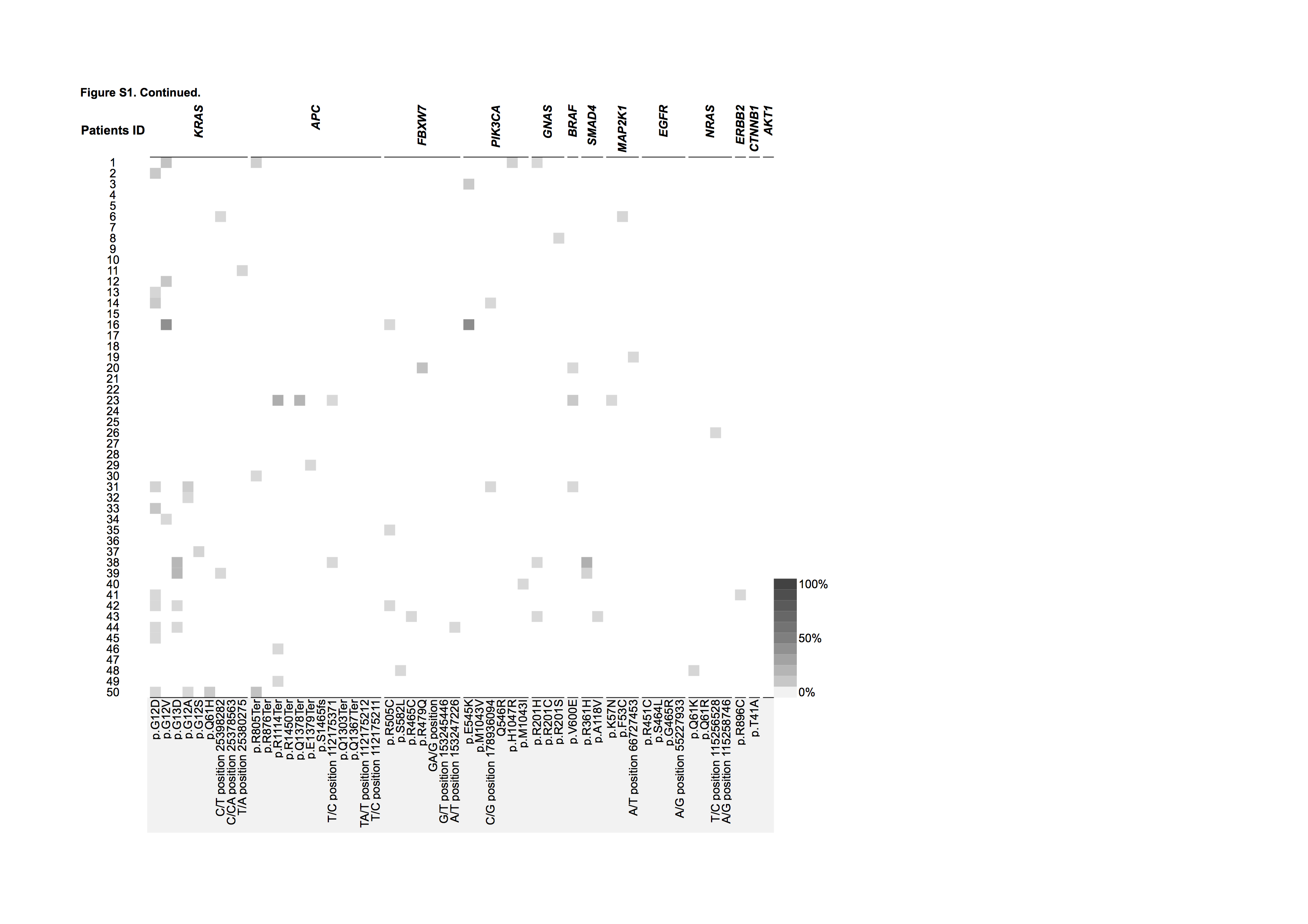

Supplement: Supplementary file 2 [file CAM4-8-408-s002.tiff]

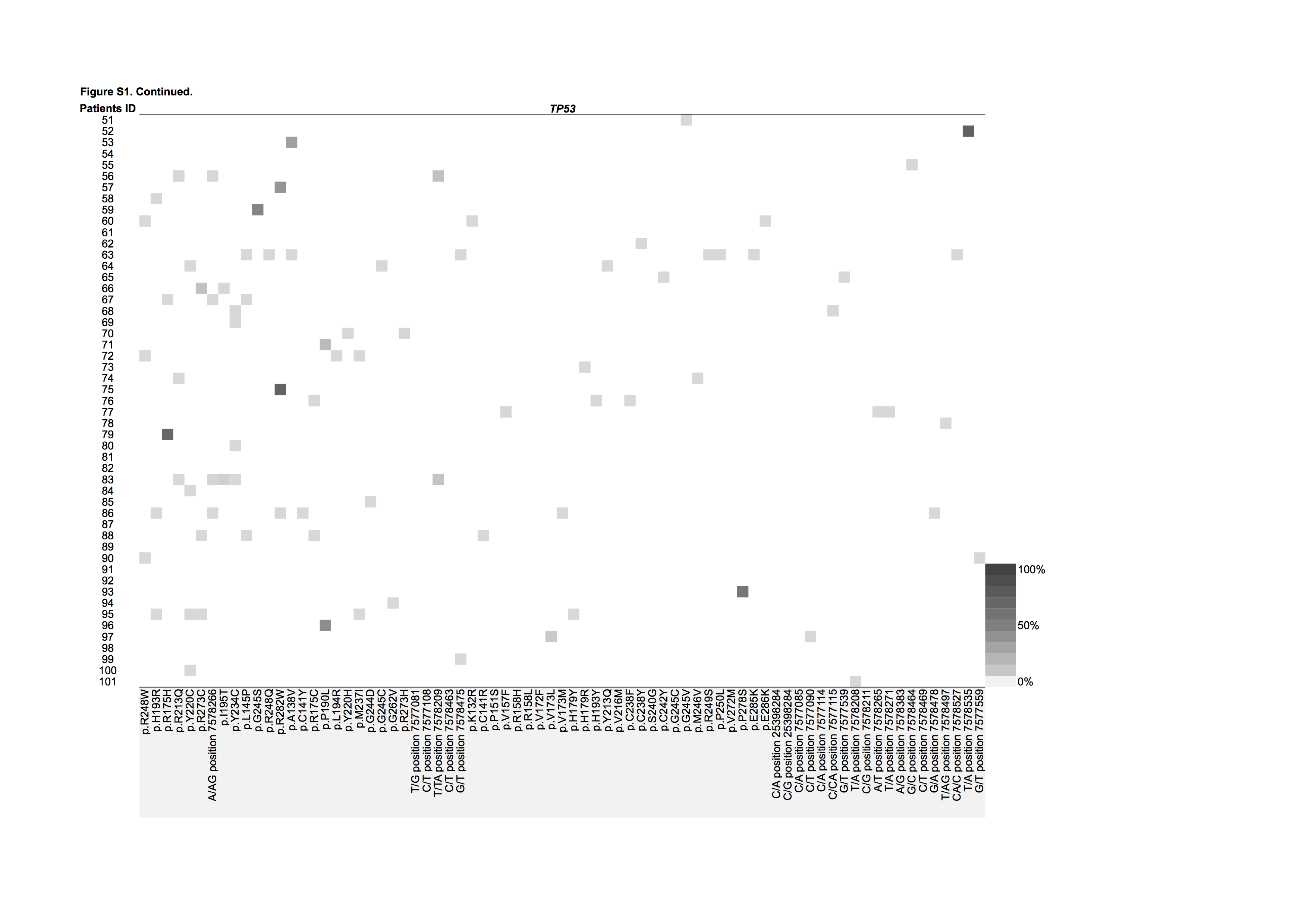

Supplement: Supplementary file 3 [file CAM4-8-408-s003.tiff]

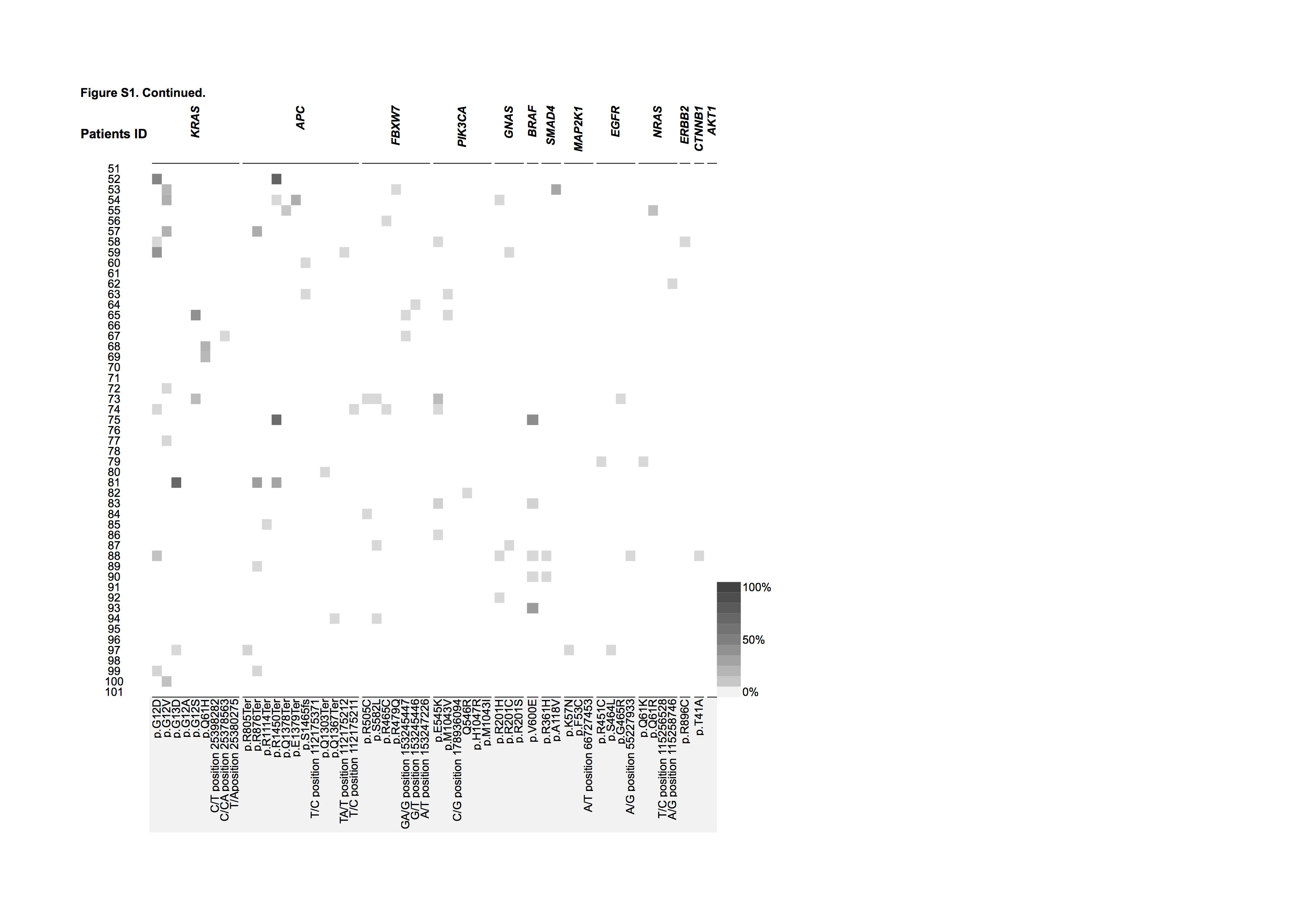

Supplement: Supplementary file 4 [file CAM4-8-408-s004.tiff]

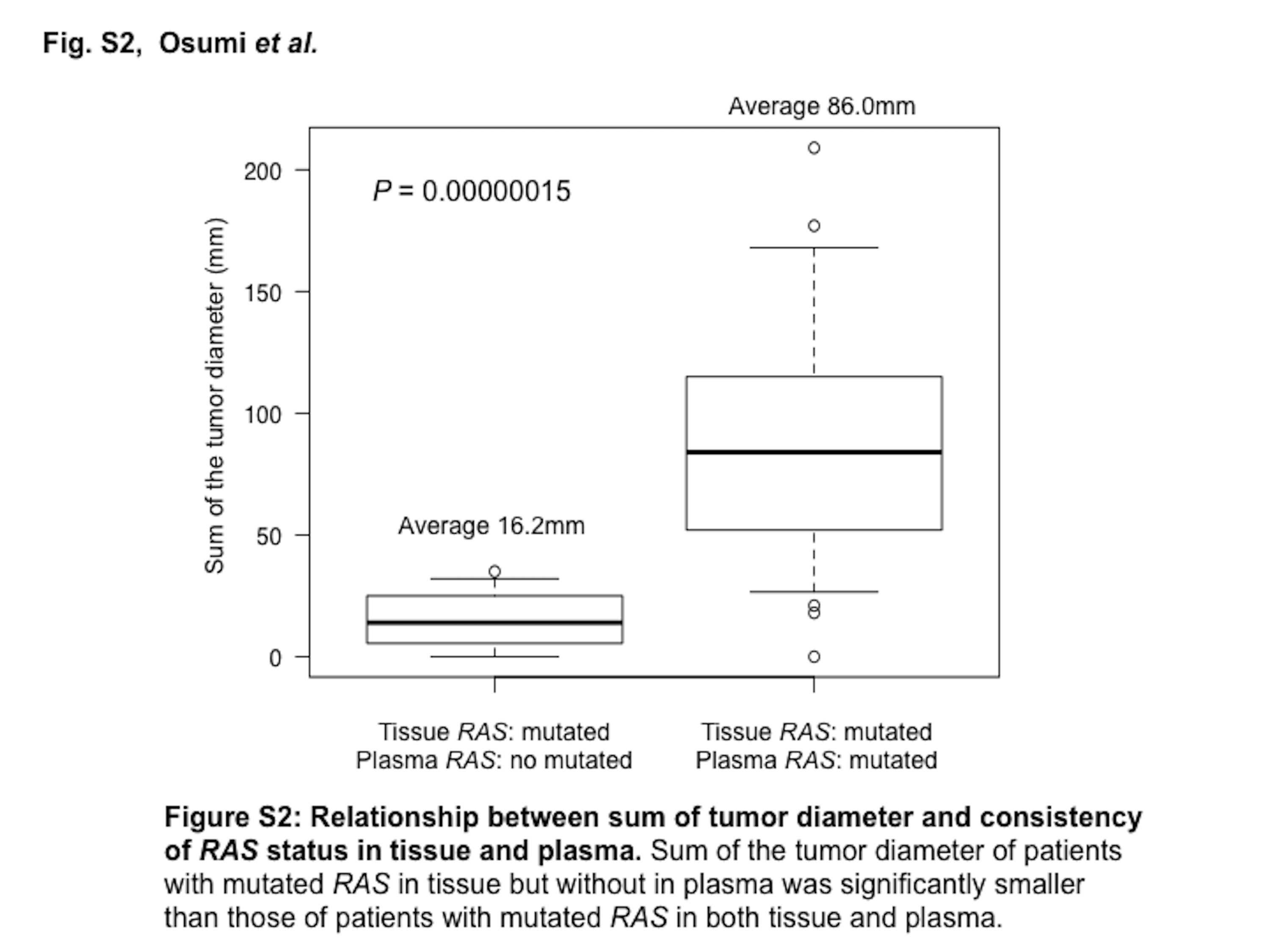

Supplement: Supplementary file 5 [file CAM4-8-408-s005.tiff]
